# Tracking the decline of weasels in North America

**DOI:** 10.1371/journal.pone.0254387

**Published:** 2021-07-21

**Authors:** David Jachowski, Roland Kays, Andrew Butler, Anne M. Hoylman, Matthew E. Gompper

**Affiliations:** 1 Department of Forestry and Environmental Conservation, Clemson University, Clemson, South Carolina, United States of America; 2 School of Life Sciences, University of KwaZulu-Natal, Pietermaritzburg, South Africa; 3 North Carolina Museum of Natural Sciences, Raleigh, North Carolina, United States of America; 4 Department of Forestry and Environmental Resources, North Carolina State University, Raleigh, North Carolina, United States of America; 5 Asombro Institute for Science Education, Las Cruces, New Mexico, United States of America; 6 Department of Fish, Wildlife and Conservation Ecology, New Mexico State University, Las Cruces, New Mexico, United States of America; Sichuan University, CHINA

## Abstract

Small carnivores are of increasing conservation concern globally, including those formerly thought to be widespread and abundant. Three weasel species (*Mustela nivalis*, *M*. *frenata*, and *M*. *erminea*) are distributed across most of North America, yet several recent studies have reported difficulty detecting weasels within their historical range and several states have revised the status of weasels to that of species of conservation concern. To investigate the status and trends of weasels across the United States (US) and Canada, we analyzed four separate datasets: historical harvests, museum collections, citizen scientist observations (iNaturalist), and a recent US-wide trail camera survey. We observed 87–94% declines in weasel harvest across North America over the past 60 years. Declining trapper numbers and shifts in trapping practices likely partially explain the decline in harvest. Nonetheless, after accounting for trapper effort and pelt price, we still detected a significant decline in weasel harvest for 15 of 22 evaluated states and provinces. Comparisons of recent and historical museum and observational records suggest relatively consistent distributions for *M*. *erminea*, but a current range gap of >1000 km between two distinct populations of *M*. *nivalis*. We observed a dramatic drop-off in *M*. *frenata* records since 2000 in portions of its central, Great Lakes, and southern distribution, despite extensive sampling effort. In 2019, systematic trail camera surveys at 1509 sites in 50 US states detected weasels at 14 sites, all of which were above 40^o^ latitude. While none of these datasets are individually conclusive, they collectively support the hypothesis that weasel populations have declined in North America and highlight the need for improved methods for detecting and monitoring weasels. By identifying population declines for small carnivores that were formerly abundant across North America, our findings echo recent calls to expand investigations into the conservation need of small carnivores globally.

## Introduction

Globally, small carnivores are increasingly recognized as a group of species in need of conservation attention [1–3]. Compared to larger carnivores, they are equally endangered with extinction, yet their conservation status is less frequently evaluated [4]. To counteract this trend there is an urgent need to assess the status and trends of small carnivores.

Members of the genus *Mustela* (= *Neogale*; [5]), hereafter referred to as weasels, were historically widespread and occurred in diverse terrestrial ecosystems across North America. Three of these species were commonly trapped furbearers: *M*. *erminea* (ermine, stoat or short-tailed weasel), *M*. *nivalis* (least weasel), and *M*. *frenata* (long-tailed weasel). *M*. *erminea* and *M*. *nivalis* are primarily northern species; the southern extent of their geographic ranges occurs in the Rocky or Appalachian Mountains, respectively [6, 7]. *M*. *frenata* ranges from Canada to Bolivia, the most extensive range of any mustelid in the western hemisphere, including historical records from all contiguous US states and Canadian provinces, and nearly all life zones [8].

Globally, weasels are considered “least concern” [9–11] and can be harvested legally across most of their North American range (Table 1). At the same time, regional recognition of harvest declines has resulted in some recent attention to their management status. Over the past decade, several states and provinces have changed their formal conservation ranking. *M*. *nivalis* has received the highest level of conservation attention to date, being listed as a species of concern or in need of conservation in 53% of states and provinces within its range, most prominently in the southern portion of the range (Table 1). *M*. *frenata* and *M*. *erminea* are listed as species of concern or in need of conservation in 24% and 10% of states and provinces within their respective ranges (Table 1).

**Table 1:** NatureServe state conservation status rank of weasels (*Mustela* sp.) in the United States and Canada (data accessed November 24, 2020).

Region	State/Province	Abbreviation	Long-tailed weasel (*Mustela frenata*)	Short-tailed weasel (*Mustela erminea*)	Least weasel (*Mustela nivalis*)
Alaska/Western Canada	Alaska	AK	NA	Furbearer (S5)	Furbearer (S4)
	British Columbia	BC	Furbearer (S5)	Furbearer (S5)	Furbearer (S4)
	Northwest Territories	NWT	NA	Furbearer (S5)	Furbearer (S5)
	Nunavut	NT	NA	Furbearer (S5)	Furbearer (S5)
	Yukon Territory	YT	NA	Furbearer (S5)	Furbearer (S4)
Mid-latitude Forests	Delaware	DE	Species of Greatest Conservation Need (S5)	NA	NA
	Illinois	IL	Furbearer (S4)	NA	Furbearer (S3)
	Indiana	IN	Furbearer (S4)	NA	Furbearer/Species of Conservation Concern (S2)
	Kentucky	KY	Furbearer (S4)	NA	Furbearer/Species of Special Concern (S2S3)
	Maryland	MD	Furbearer (S5)	NA	In Need of Conservation (S2S3)
	Missouri	MO	Furbearer (closed season)/Species of Conservation Concern (S3)	NA	Furbearer (closed season)/Species of Conservation Concern (S3)
	New Jersey	NJ	Furbearer (S5)	Furbearer (SU)	NA
	North Carolina	NC	Furbearer (S3)	NA	Furbearer/Significantly Rare-Game (S2)
	Ohio	OH	Furbearer (SNR)	Furbearer/Species of Concern (S3)	Furbearer (SNR)
	Tennessee	TN	Furbearer (S5)	NA	Furbearer/Species of Greatest Conservation Need (S2)
	Virginia	VA	Furbearer (S5)	NA	Furbearer (S3)
	West Virginia	WV	Furbearer (S4)	NA	Furbearer (S3)
North Central	Iowa	IA	Furbearer/ Species of Greatest Conservation Need (S4)	Furbearer/ Species of Greatest Conservation Need (S4)	Furbearer/Species of Greatest Conservation Need (S3)
	Kansas	KS	Furbearer (S2S3)	NA	Furbearer (S4)
	Manitoba	MB	Furbearer (S3)	Furbearer (S5)	Furbearer (S3S4)
	Nebraska	NE	Furbearer/Species of Greatest Conservation Need (S2)	NA	(S5)
	North Dakota	ND	Furbearer (SNR)	Furbearer (SNR)	Furbearer (SNR)
	Saskatchewan	SN	Furbearer (S5)	Furbearer (S5)	Furbearer (S5)
	South Dakota	SD	Furbearer (S5)	Furbearer (S4)	Furbearer (S5)
Northern Forest	Connecticut	CT	Furbearer (S5)	Furbearer (S5)	NA
	Maine	ME	Furbearer (S5)	Furbearer (S5)	NA
	Massachusetts	MA	Furbearer (S5)	Furbearer (S5)	NA
	Michigan	MI	Furbearer (S5)	Furbearer (S5)	Furbearer (S5)
	Minnesota	MN	Furbearer (SNR)	Furbearer (SNR)	Species of Special Concern (S3)
	New Brunswick	NB	Furbearer (S5)	Furbearer (S5)	NA
	Newfoundland and Labrador	NL	NA	Furbearer (S4S5)	Furbearer (S1S3)
	New Hampshire	NH	Furbearer (S5)	Furbearer (S5)	NA
	New York	NY	Furbearer (S5)	Furbearer (S5)	Furbearer/Species of Potential Conservation Need (S1)
	Nova Scotia	NS	NA	Furbearer (S5)	NA
	Ontario	ON	Furbearer (S4)	Furbearer (S5)	Furbearer (SU)
	Pennsylvania	PA	Furbearer (S5)	Furbearer (S5)	Furbearer (S5)
	Prince Edward Island	PE	NA	Furbearer (S5)	NA
	Quebec	QC	Furbearer (S5)	Furbearer (S5)	Furbearer (S2S3)
	Rhode Island	RI	Furbearer (S4)	Furbearer (SH)	NA
	Vermont	VT	Furbearer/Species of Greatest Conservation Need (S3S4)	Furbearer (S5)	NA
	Wisconsin	WI	Furbearer (S4)	Furbearer (S4)	Furbearer (SU)
Rockies	Alberta	AA	Furbearer (S3S4)	Furbearer (S5)	Furbearer (S5)
	Arizona	AZ	Furbearer/Species of Greatest Conservation Need (S4)	NA	NA
	Colorado	CO	Furbearer (S5)	Furbearer (S4)	NA
	Idaho	ID	Predatory Wildlife (S5)	Predatory Wildlife (S4)	NA
	Montana	MT	Predatory Animal (S5)	Predatory Animal (S5)	Predatory Animal (S4)
	Nevada	NV	Furbearer (S5)	Furbearer (S3)	NA
	New Mexico	NM	Furbearer (S4)	Furbearer (S3)	NA
	Utah	UT	Furbearer (S4)	Furbearer (S3S4)	NA
	Wyoming	WY	Furbearer (S5)	Furbearer (S5)	Furbearer/Species of Greatest Conservation Need (S1S2)
South	Alabama	AL	Non-game/Species of Greatest Conservation Need (S3)	NA	NA
	Arkansas	AR	Furbearer/Species of Greatest Conservation Need (S3)	NA	NA
	Florida	FL	Furbearer (S5)	NA	NA
	Georgia	GA	Furbearer (S5)	NA	Furbearer/Species of Greatest Conservation Need (S1)
	Louisiana	LA	Species of Greatest Conservation Need (S3)	NA	NA
	Mississippi	MS	Furbearer/Species of Greatest Conservation Need (S2)	NA	NA
	Oklahoma	OK	Furbearer/Species of Greatest Conservation Need (S2)	NA	NA
	South Carolina	SC	Furbearer (S3)	NA	Furbearer (S3)
	Texas	TX	Species of Greatest Conservation Need (S5)	NA	NA
West Coast	California	CA	Nongame (SNR)	Nongame (SNR)	NA
	Oregon	OR	Unprotected Mammal (S5)	Unprotected Mammal (S5)	NA
	Washington	WA	Furbearer (S5)	Furbearer (S5)	NA

NatureServe ranks are S1 = critically imperiled, S2 = imperiled, S3 = vulnerable, S4 = apparently secure, S5 = secure, SNR = unranked, SU = unrankable (see https://explorer.natureserve.org/AboutTheData/Statuses). The terms “Furbearer,” “Furbearer with closed season,” “Predatory Animal,” and “Predatory Wildlife” implies that the species is considered a game or nuisance species within that state or province, although regulations on harvest vary among states and provinces. The terms “Species of Conservation Concern,” “Species of Greatest Conservation Need,”and “Species of Potential Conservation Need” are designations from the corresponding state’s State Wildlife Action Plan.

We have limited knowledge of the current abundance and distribution of all three weasel species in North America. Weasels can be monitored through live traps as well as noninvasive survey techniques such as snow tracking, baited track plates and tunnels, hair snares, or trail cameras [12–15], but these approaches vary in their efficacy. For example, in a comparison of noninvasive survey techniques for carnivores in the Adirondack Mountains of New York, track plates were most effective for detecting weasels, but probability of detection was low (<10%; [16]). Further, apart from a few regional carnivore surveys [e.g., 16, 17], there have been few large-scale quantitative assessments of their status [but see 18]. Finally, we note their conspicuous absence or rarity in recent systematic surveys within their geographic range in the southeast and south-central US [14, 19, 20].

Recent concerns over possible declines in weasel populations highlight the need to evaluate the available data on weasel population changes critically. Toward this goal, we assessed large-scale trends in weasel populations across North America using trapping, museum, citizen science, and trail camera records. Our approach was to assess for evidence of trends suggestive of decreases in weasel numbers, and where patterns were detected, determine whether there was evidence that those trends were taxon-specific and regionally constrained. Although these data sets are imperfect, they offer insights into changes in weasel populations through broad spatio-temporal comparisons.

## Materials and methods

We surveyed four data sources for weasel records across North America: furbearer harvest records, museum specimen data (accessed through the Global Biodiversity Information Facility, GBIF; www.gbif.org), citizen science photographic data reported in iNaturalist (iNat, https://www.inaturalist.org/), and a recent nation-wide standardized trail camera survey conducted in the US (Snapshot USA, [21]).

### Harvest data

Weasels in North America have been harvested by humans for centuries for both economic and cultural reasons [22]. Fur harvest data are available for the US and Canada for nearly a century, providing one of a very few resources for tracking population-level patterns in weasel abundance and distribution [23]. Generally, agency records do not differentiate between species of *Mustela*, although some exceptions and range limits allowed us to assume the species comprising all or virtually all of the data. We started with historical data for each US state and Canadian province between the early 1900s and 1982 [24]. We supplemented this dataset with harvest information for 1970 to 2017 from reports by the Association of Fish and Wildlife Agencies [25] and with data derived from online or published reports from several states [26–29]. We then sent this dataset to the furbearer biologist of each continental state and province to request verification of harvest data, if available. The request also sought historical information on pelt price, trapper numbers and season length. Personnel from 34 state or provincial agencies responded to this inquiry; some were able to provide extensive additional data, while others simply acknowledged that there were few or no data available that could further expand the dataset.

**Fig 1:**
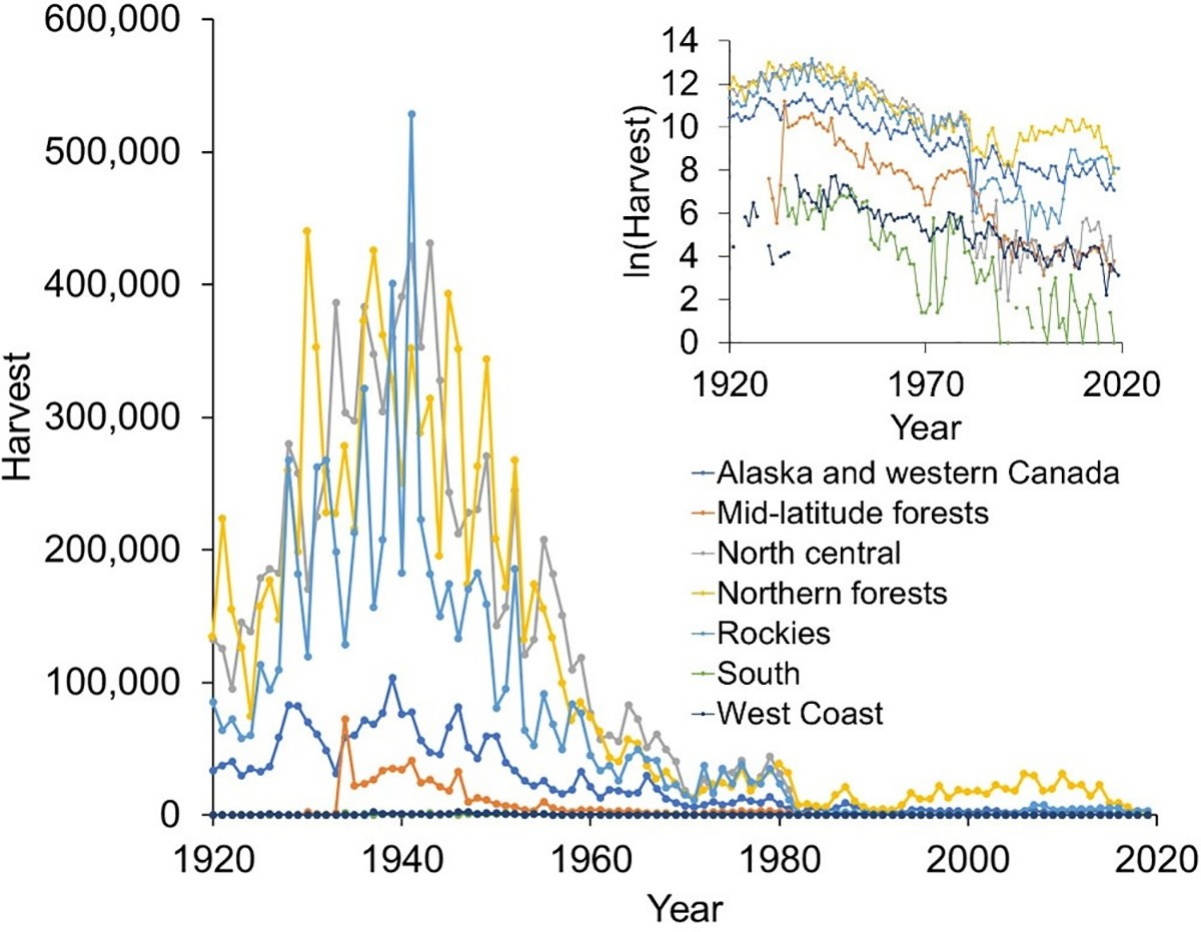
Temporal trends in the annual weasel harvest and natural log-transformed annual weasel harvest (inset) from seven regions within the United States and Canada between 1919 and 2019.

For each state and province, we then compared the available harvest data sources ([24, 25], additional state or province-specific reports, and data provided by furbearer biologists) for potential discrepancies. These discrepancies might have occurred for various reasons, most prominently due to recording or rounding errors as well as differences in how harvest was calculated in any given year. Where no conflicts in reports of an annual harvest existed, we accepted the value for that year. Where a conflict was noted and was not due to a clearly correctable recording error, we selected the most likely value for the year based on the assumption that agency-reported data were less likely to contain an error. For analysis, we excluded years with zero harvest because this value sometimes indicated that no animals were harvested, and other times indicated that the state did not record harvest numbers for that year. The length and completeness of harvest data varied considerably, with data for provinces generally beginning in 1919 and data for most states beginning in the 1930s or 1940s. Three states (Arizona, Florida, Louisiana) had little or no harvest data and were omitted from further analyses, resulting in a final harvest time-series dataset for 46 states and 12 provinces. We plotted annual harvest data (and natural log-transformed harvest data) for states and provinces within each North American ecoregion [30]. Harvest of weasels is known to have declined rapidly near the middle of the twentieth century ([31]; Fig 1). Therefore, to assess overall trends across our harvest dataset, we partitioned data as pre- and post-1960 to compare the relative difference in annual harvest rates between these two periods.

Numerous approaches have been used to control or correct for trapping effort in evaluating trends in furbearer harvest data across localities and over time, primarily involving the use of trapper numbers, season length, and pelt price [25, 32–34]. Although we attempted to collect data on these measures, only eight states and provinces (California, Minnesota, North Dakota, Newfoundland, New Hampshire, Nova Scotia, Ohio, Utah) provided at least two decades of continuous data without a gap >3 years on pelt price and trapper numbers. Four states and provinces (Arkansas, Northwest Territories, South Dakota, West Virginia) provided at least two decades of continuous data on only pelt price. Ten states continuously reported only trapper numbers (Connecticut, Kansas, Michigan, Missouri, Nebraska, Nevada, North Carolina, Oregon, Rhode Island, Vermont). Only one state (North Dakota) provided continuous data on pelt price, trapper numbers and season length simultaneously for more than five years (S1 Fig).

Pelt price, in particular, has been used widely as an indicator of trapper effort in studies of harvest trends in multiple furbearer species [33, 35, 36]. In a recent range-wide analysis of muskrat (*Ondatra zibethicus*) harvest where pelt price was only occasionally reported by states, Ahlers & Heske [37] found that pelt price was similar across localities and thus an average value could be applied to all states and provinces to correct for trapping effort. We converted Canadian weasel pelt prices to US dollar amounts using a year-specific conversion rate and corrected the pelt price for inflation using the Consumer Price Index (CPI; www.bls.gov/data/) adjusted to 2017 US dollar values. We checked for correlation using three subsets of data that had the most consistent reporting: four states and provinces from 1939–1966, five states and provinces from 1957–1985, and five states and provinces from 1984–2017. Average correlations in our subsets of data were 0.60, 0.42, and 0.23, respectively. Therefore, we did not average annual weasel pelt price across states and provinces, and instead focused on investigations into patterns of harvest within the individual states and provinces that had pelt price information. Because harvest rates within a year can be influenced by pelt price from the prior year [37], we also evaluated an effect of the previous year’s pelt price (lagPelt) on weasel harvest.

Despite its wide-scale use in harvest analyses, pelt price alone is not a consistent indicator of trapper activity [38, 39]. Trapper numbers provide a potentially more direct measure from which to control for the influence of trapper effort on weasel harvest. When the length of trapping seasons varies, season length is also often taken into account when attempting to control for trapper effort (e.g., number of trappers/days in a trapping season; [32]). For states and provinces that reported season length (n = 15), the length varied among states and provinces but was relatively consistent within individual states and provinces over time. Ten states and provinces (Alberta, Maine, Missouri, Nebraska, Northwest Territories, Ohio, Oregon, Rhode Island, Virginia, Washington) reported that trapping season length remained the same, two states (Kansas, North Dakota) reported an increase, and four states (Minnesota, New Hampshire, West Virginia, Wyoming) reported that trapping season length decreased over time. Three of the states that reported a decrease in season length (Minnesota, New Hampshire, West Virginia) went from no closed season to a winter trapping season only—a change that was unlikely to affect trapping greatly given that weasels typically are captured during winter months. Overall, given how infrequently season length was reported and that when reported, it typically did not change over time within states or provinces, we chose to exclude season length in our analysis and instead focus on trapper numbers. For each state or province where consistent trapper number data were available (n = 18), we also calculated per-trapper weasel harvest (AdjHarvest).

We used linear regression to evaluate the influence of year, pelt price and number of trappers on annual weasel (all species combined) harvest and per-trapper harvest within each state or province that consistently (i.e., 20 years of continuous data with no gaps >3yrs) reported harvest along with either pelt price or trapper number (or both) annually. For states and provinces that consistently reported both trapper numbers and pelt prices (California, Minnesota, North Dakota, Newfoundland, New Hampshire, Nova Scotia, Ohio, Utah), we fit a model containing year, natural log-transformed pelt price, and natural log-transformed trapper number (Harvest ~ Year + Pelt + Trapper), as well as a model containing year, natural log-transformed pelt price of the previous year, and natural log-transformed trapper number (Harvest ~ Year + lagPelt + Trapper). We did not include Pelt and lagPelt in the same model as the correlation between the two variables was greater than 0.60 across states and provinces. For states and provinces that consistently reported only harvest and pelt price (Arkansas, Northwest Territories, South Dakota, West Virginia), we fit a model that included natural log-transformed pelt price (Harvest ~ Year + Pelt) and a model that included the natural log-transformed pelt price of the previous year in the dataset (Harvest ~ Year + lagPelt). For states and provinces that only consistently reported harvest and trapper number (Connecticut, Kansas, Michigan, Missouri, Nebraska, Nevada, North Carolina, Oregon, Rhode Island, Vermont), we fit a model that included year and natural log-transformed trapper number (Harvest ~ Year + Trapper). For states and provinces for which we were able to calculate per-trapper weasel harvest (AdjHarvest) for use as a response variable, we similarly evaluated the effect of year, pelt price and pelt price from the previous year. We interpreted beta estimates that did not have 95% confidence intervals overlapping zero as indicating that a predictor variable had either a significant positive or negative effect on weasel harvest. Apart from Pelt and lagPelt, we found no strong correlations among predictive variables (<0.60). All variables were standardized to a mean of zero and a standard deviation of 1 for analysis.

### Biodiversity databases: Museums and iNaturalist

We searched GBIF for all museum records from the US, Canada and Mexico that were based on museum specimens [40]. Although some iNaturalist records are also available in GBIF, we obtained records directly from the iNaturalist website because it includes copyrighted observations not sent to GBIF. We considered only research-grade observations from the iNaturalist platform that included a photograph voucher and were identified to species. To investigate changes in distribution, we used both the museum data, which have more historical records, and the iNaturalist data, which have more recent (since 2000) records, and mapped records before and after the year 2000. To investigate change in the distribution of *M*. *frenata* more closely, we compared its occurrence in ecoregions before and since 2000, limiting our inference to those 57 regions that had at least ten records before 2000.

### National trail camera survey

We used the 2019 Snapshot USA dataset as a systematic assessment of the present distribution of weasels [21]. This survey consisted of trail cameras deployed at 1509 sites across 110 arrays in all 50 states, for a total of 53,505 trap nights of effort. All cameras were set ~50cm above ground, without bait, in September and October of 2019. The number of cameras per array varied from 4 to 49 (mean = 13.7) and were set 300-5000m apart. A variety of camera models were used, but all had an infrared flash and a relatively fast (<0.5sec) trigger time.

## Results

### Harvest data

Between 1919 and 2019, >31.5 million weasels were harvested across 58 states and provinces (Fig 1). As data were incomplete across years and states, this number represents a minimum estimate of the actual harvest during this time. Average annual harvest (mean = 312,246; SE = 36,009) over the 101-year time span of our trapping records varied greatly among years and regions but generally declined across all regions over time (Fig 1). When averaged across North American ecoregions, mean weasel harvest declined 74–94% between 1920–1960 vs. 1961–2019 (Table 2). Declines were most dramatic in northeastern (92.8%) and north-central (93.7%) North America (Table 2). For states or provinces where species could be discerned either because only one species occurs (e.g., *M*. *frenata* in AR), because the vast majority of harvested weasels are likely from one species (e.g., *M*. *frenata* in MO), or because the state or province tracked species individually (e.g., *M*. *frena*ta and *M*. *erminea* in MN), these patterns were seen for both *M*. *frenata* and *M*. *erminea* (no state or province monitored *M*. *nivalis* distinctly from other weasels, and no state is inhabited solely by *M*. *nivalis*).

**Table 2:** Average number (and standard error) of weasels harvested annually in the United States and Canada from 1919 to 1960 and from 1961 to 2019.

Ecoregion	Average harvest 1919–1959	Average harvest 1960–2018	Percent decline from pre1960
Alaska and western Canada	49,193 (3,387)	6,400 (773)	87.0
Mid-latitude Forests	12,130 (2,443)	879 (146)	92.7
North Central	231,428 (15,237)	14,583 (2,980)	93.7
Northern Forests	227,585 (15,832)	20,517 (1,766)	91.0
Rockies	153,110 (15,637)	11,863 (1,921)	92.2
South	384 (66)	43 (11)	88.8
West Coast	578 (99)	146 (15)	74.8

See Table 1 for a breakdown of states and provinces within each ecoregion.

**Fig 2:**
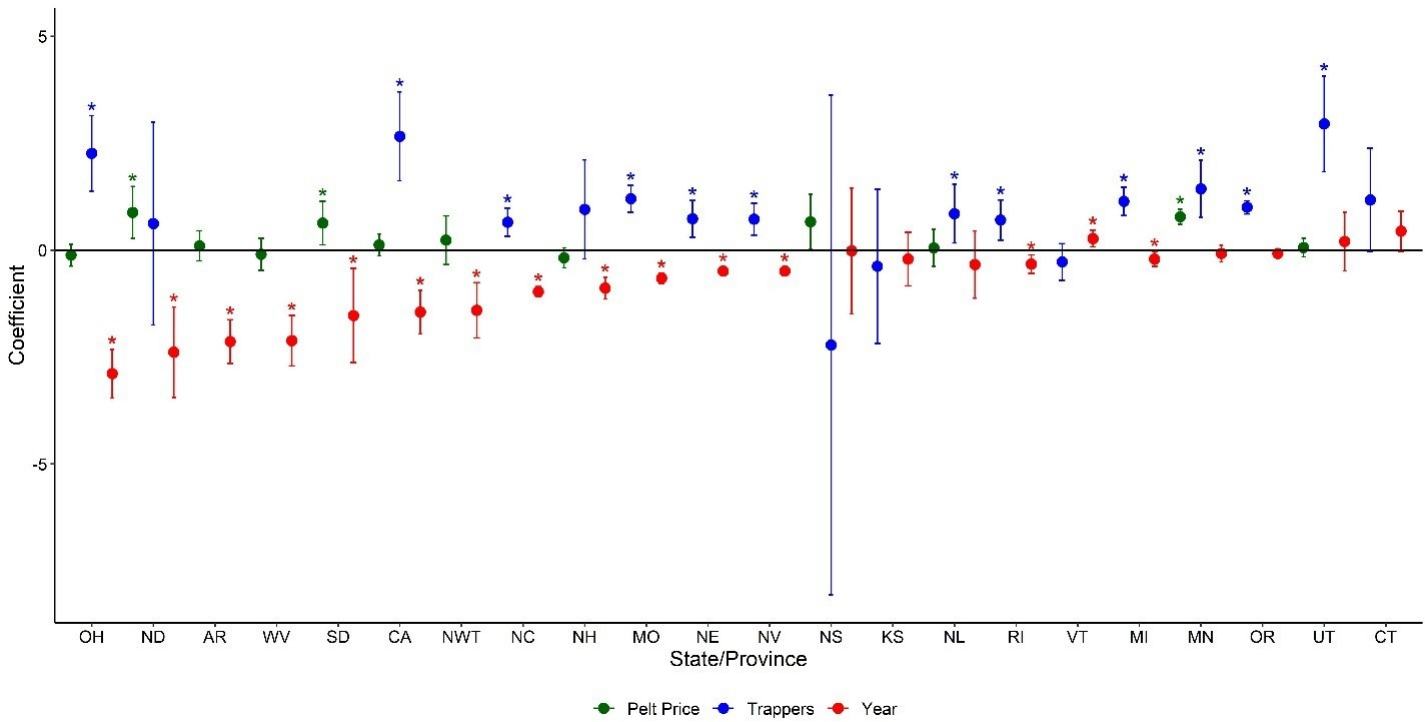
Analysis of factors associated with annual trends in weasel harvest from 1920–2017 for 22 states or provinces in North America (harvest ~ year + pelt, harvest ~ year + pelt + trappers, harvest = year + trappers). Markers represent beta estimates and 95% confidence intervals of the effect of year, current-year pelt price, and number of trappers from linear regression models. Asterisks indicate significant positive and negative effects on harvest. See Table 1 for state/province abbreviations.

**Fig 3:**
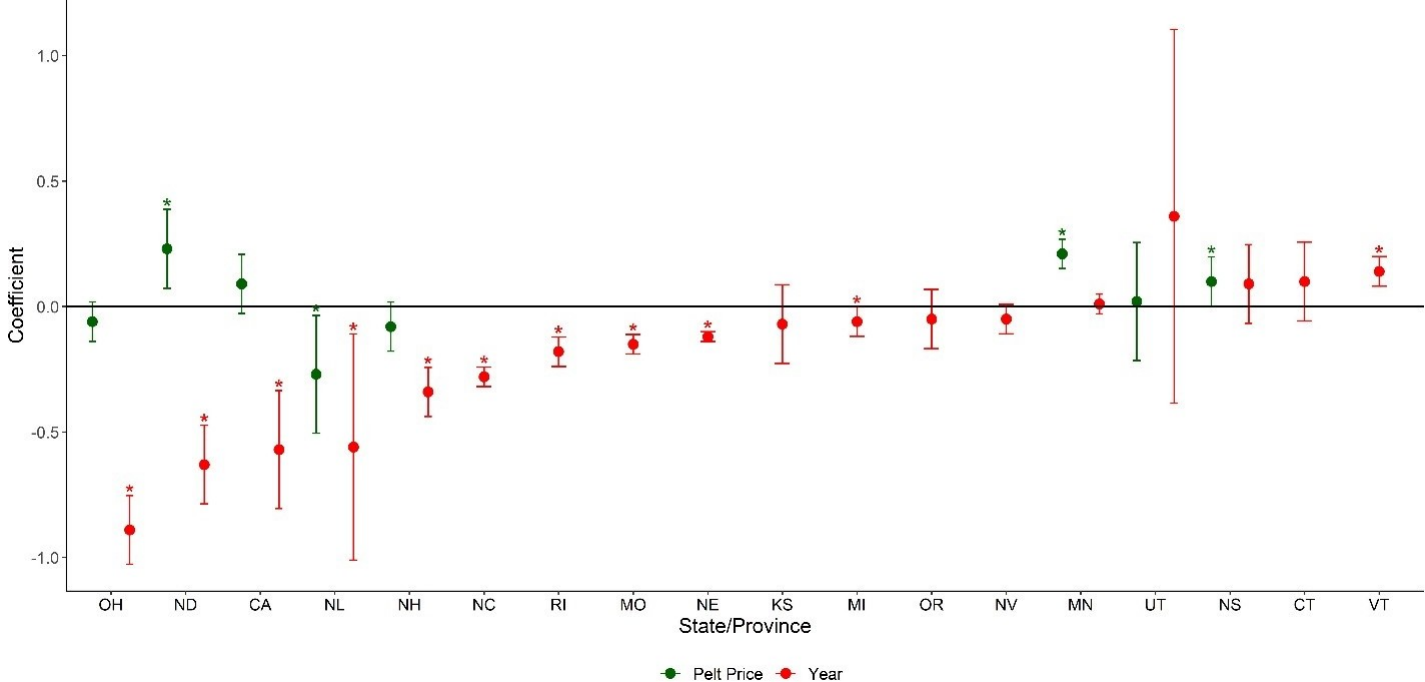
State- and province-specific analysis of pelt price and/or year on historical per capita weasel harvest (adjHarvest = Yr + Pelt, adjHarvest = Yr) in North American states and provinces for which >20 years of continuous data were available between 1919 and 2017 (see methods section). Markers represent beta estimates and 95% confidence intervals of year and current-year pelt price from linear regression models. Asterisks indicate significant positive and negative effects on harvest. See Table 1 for state/province abbreviations.

After accounting for changes in trapper effort, we found a significant negative effect of year on weasel harvest in 64% (14 of 22) of states and provinces, suggesting that harvests were declining more than expected based on changes in trapper effort alone. We found a significant positive effect of year (i.e., increasing harvest rate over time) for one state (Vermont) and no significant effect of year for seven states and provinces (Fig 2). We found a significant positive effect of pelt price on harvest in 25% (3 of 12) of states and provinces we were able to evaluate (Fig 2), and a significant negative effect of pelt price in none of the states and provinces (Fig 2). We found a significant positive effect of the number of trappers on harvest in 67% (12 of 18) of states and provinces for which this could be evaluated, but found no effect of trapper numbers on harvest in the remaining 33% (Fig 2). When substituting pelt price from the previous year (lagPelt) for current-year pelt price, we observed no change in variables found to be significant nor in the direction of relationships within our models, with the exception of a significant positive effect of pelt price on weasel harvest for Newfoundland, for which there also was a significant positive relationship between current-year pelt price (S1 Table). Similar to annual harvest analysis, we found no effect of pelt price on per-trapper weasel harvest (AdjHarvest) in 50% (4 of 8) of states and provinces for which we were able to evaluate it, and a significant negative effect of year on per-trapper weasel harvest (AdjHarvest) in 56% (10 of 18) of states and provinces (Fig 3). The effect of year remained similar across states and provinces when using the pelt price of the previous year (S1 Table).

### Biodiversity databases: Museums and iNaturalist

We obtained 12,816 records of *M*. *nivalis*, *M*. *frenata*, and *M*. *erminea* from 61 museum collections that were represented by voucher specimens and 1,509 from iNaturalist that were judged as research grade. Most (89%) museum data were from specimens collected prior to the year 2000, whereas nearly all (99%) iNaturalist data were collected post-2000 (Table 3). Prior to 2000, museum specimens of *M*. *frenata* and *M*. *erminea* were similar in abundance (46% each), but *M*. *erminea* comprised 75% of specimens collected after 2000. This bias towards *M*. *erminea* was not mirrored in the iNaturalist data, for which 67% of observations were of *M*. *frenata*. Across both museum and iNaturalist datasets, *M*. *nivali*s comprised ≤ 10% of weasel specimens or observations.

**Table 3:** Number of records (and proportion) by species for North American weasels archived as specimens in museums and as photographs in iNaturalist before and after the year 2000.

Data Type	Time Period	*Mustela erminea*	*Mustela frenata*	*Mustela nivalis*
Museum	Before 2000	5240 (46%)	5269 (46%)	945 (8%)
Museum	2000 and after	1028 (75%)	193 (14%)	141 (10%)
	total	6268 (49%)	5462 (43%)	1086 (8%)
iNat	Before 2000	3	3	-
iNat	2000 and after	455 (30%)	1009 (67%)	39 (3%)
	total	458 (30%)	1012 (67%)	39 (3%)

**Fig 4:**
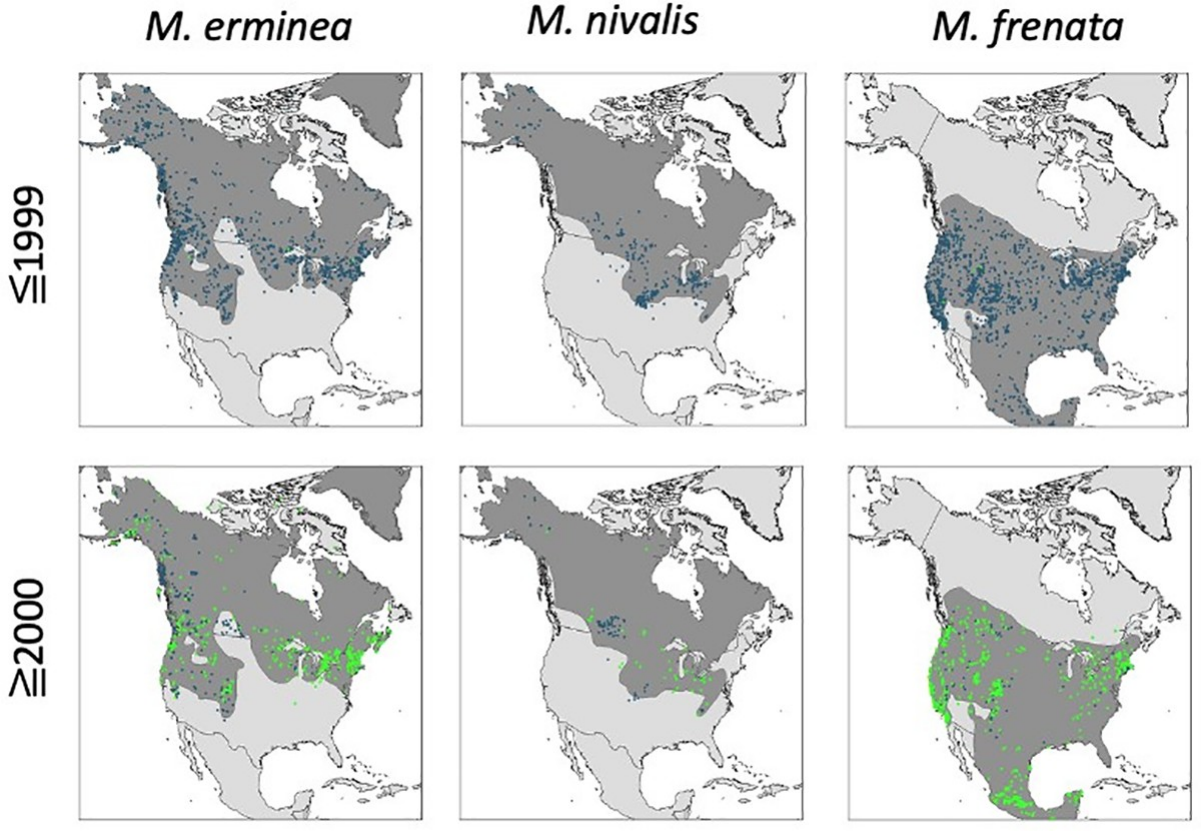
Distribution of records for three *Mustela* species from museum specimens (blue dots) and iNaturalist citizen science observation (green squares) from two time periods. Dark gray shading shows the range map for each species.

Museum specimen locality records for *M*. *erminea* and *M*. *nivalis* show relatively similar patterns before and after 2000 (Fig 4). There are relatively sparse records in the far north and a handful of records outside of the International Union for the Conservation of Nature (IUCN) existing range that might justify small range extensions for both species [9, 10]. In both time periods, there is a large gap in *M*. *nivalis* records between Alaska and southern Canada that suggests a >1000 km gap in distribution not presently reflected by the IUCN range map.

**Fig 5:**
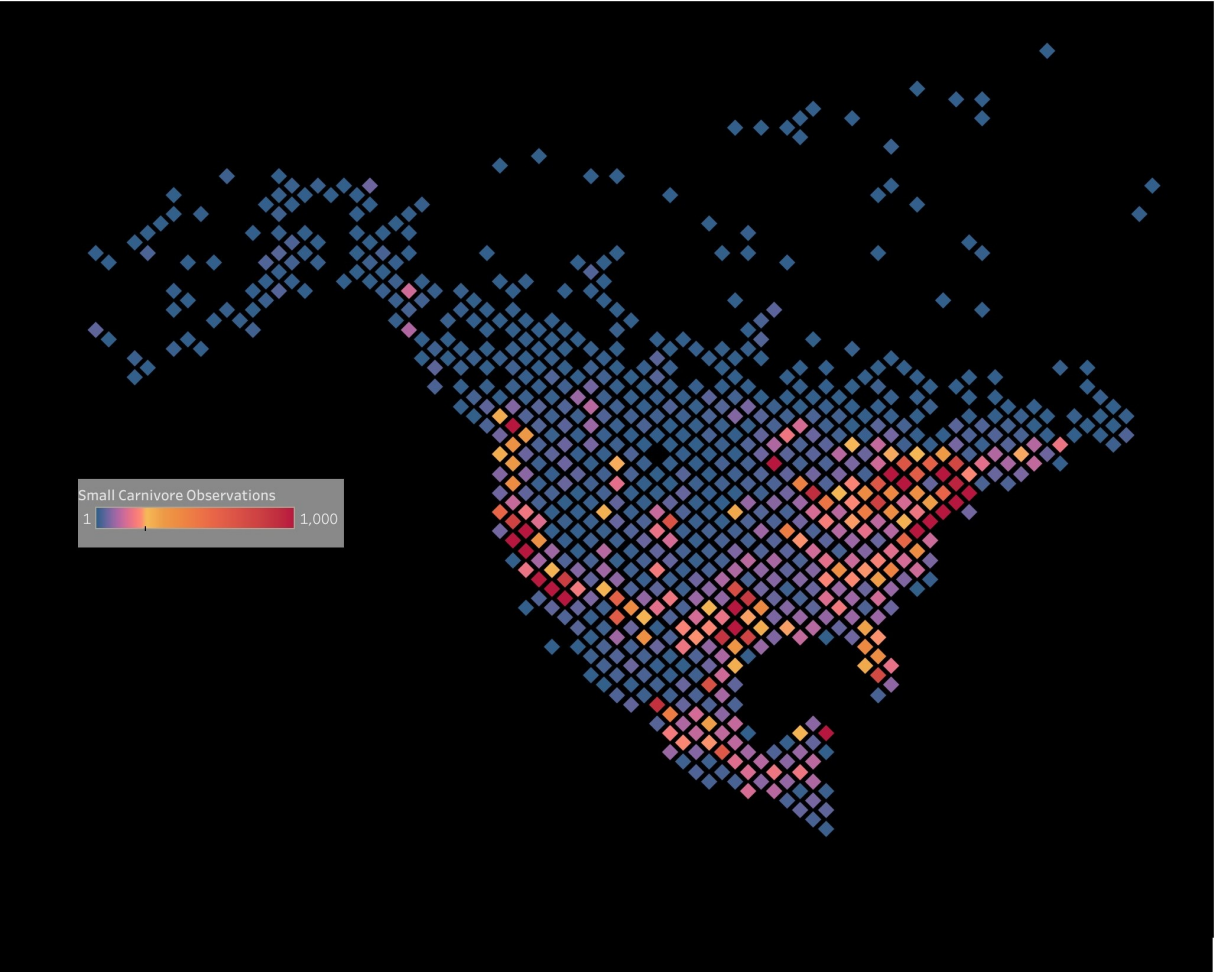
Density map of 83,981 iNaturalist records of small carnivores recorded since 2000 in North America.

In contrast, the temporal comparison of pre- and post-2000 records of *M*. *frenata* reveal striking differences. Large areas with frequent records pre-2000 lacked records post-2000. The lack of recent records from some areas is likely not an artifact of limited sampling as many tens of thousands of records of other small carnivores such as raccoon (*Procyon lotor*), striped skunk (*Mephitis mephitis*), ringtail (*Bassariscus astutus*) and mink (*Mustela vison*) have been posted to iNaturalist from across the region, including areas with few *M*. *frenata* records (Fig 5).

All but one region had fewer records of museum specimens and iNaturalist observations combined for *M*. *frenata* since 2000, averaging 73% fewer, which is unsurprising given the shorter time interval (Table 3). Specifically, there were no or very few records of *M*. *frenata* since 2000 in the eastern coastal plain, the central forest-grassland transition, and Great Lakes forests despite having dozens of records before 2000 (Fig 4, Table 4). Six western ecoregions also showed a greater than average decline in *M*. *frenata* records. Regions with more than average records since 2000 include mountainous forests around the continent and cold weather grasslands and shrub habitats.

**Table 4:** Change in the number of museum and iNaturalist records of *Mustela frenata* per ecoregion before 2000 and from 2000 and after that year.

Ecoregion	Records before 2000	Records since 2000	Difference	Proportional change
Central and Southern mixed grasslands	24	0	-24	-1.00
Central tall grasslands	24	0	-24	-1.00
Flint Hills tall grasslands	18	0	-18	-1.00
Northern tall grasslands	15	0	-15	-1.00
Mississippi lowland forests	10	0	-10	-1.00
Southeastern mixed forests	56	1	-55	-0.98
Central forest-grasslands transition	183	6	-177	-0.97
Western Great Lakes forests	30	1	-29	-0.97
Wyoming Basin shrub steppe	56	2	-54	-0.96
Nebraska Sand Hills mixed grasslands	23	1	-22	-0.96
Okanagan dry forests	45	2	-43	-0.96
Eastern Cascades forests	36	2	-34	-0.94
Blue Mountains forests	17	1	-16	-0.94
Middle Atlantic coastal forests	16	1	-15	-0.94
Peten-Veracruz moist forests	14	1	-13	-0.93
Southern Great Lakes forests	285	21	-264	-0.93
California Central Valley grasslands	40	3	-37	-0.93
Central U.S. hardwood forests	25	2	-23	-0.92
Southeastern conifer forests	32	3	-29	-0.91
Allegheny Highlands forests	67	7	-60	-0.90
Central Pacific coastal forests	75	8	-67	-0.89
Atlantic coastal pine barrens	22	3	-19	-0.86
Western short grasslands	79	11	-68	-0.86
Great Basin montane forests	13	2	-11	-0.85
Klamath-Siskiyou forests	19	3	-16	-0.84
Chihuahuan desert	67	11	-56	-0.84
Cascade Mountains leeward forests	12	2	-10	-0.83
Upper Midwest forest-savanna transition	41	7	-34	-0.83
Arizona Mountains forests	76	13	-63	-0.83
Central American pine-oak forests	19	4	-15	-0.79
California coastal sage and chaparral	200	57	-143	-0.72
Palouse grasslands	24	7	-17	-0.71
California montane chaparral and woodlands	40	12	-28	-0.70
Alberta Mountain forests	10	3	-7	-0.70
South Central Rockies forests	65	20	-45	-0.69
Northeastern coastal forests	163	52	-111	-0.68
Wasatch and Uinta montane forests	72	24	-48	-0.67
Colorado Plateau shrublands	71	24	-47	-0.66
Northern short grasslands	58	20	-38	-0.66
New England-Acadian forests	66	25	-41	-0.62
Montana Valley and Foothill grasslands	15	6	-9	-0.60
Canadian Aspen forests and parklands	24	10	-14	-0.58
Eastern Great Lakes lowland forests	42	18	-24	-0.57
Great Basin shrub steppe	107	46	-61	-0.57
Sierra Nevada forests	75	34	-41	-0.55
Eastern forest-boreal transition	21	10	-11	-0.52
Snake-Columbia shrub steppe	46	22	-24	-0.52
North Central Rockies forests	39	19	-20	-0.51
Appalachian-Blue Ridge forests	39	20	-19	-0.49
Puget lowland forests	58	30	-28	-0.48
Central and Southern Cascades forests	12	7	-5	-0.42
California interior chaparral and woodlands	201	121	-80	-0.40
Colorado Rockies forests	114	70	-44	-0.39
Willamette Valley forests	16	10	-6	-0.38
Northern mixed grasslands	23	17	-6	-0.26
Northern California coastal forests	35	32	-3	-0.09
Appalachian mixed mesophytic forests	10	11	1	0.10

**Fig 6:**
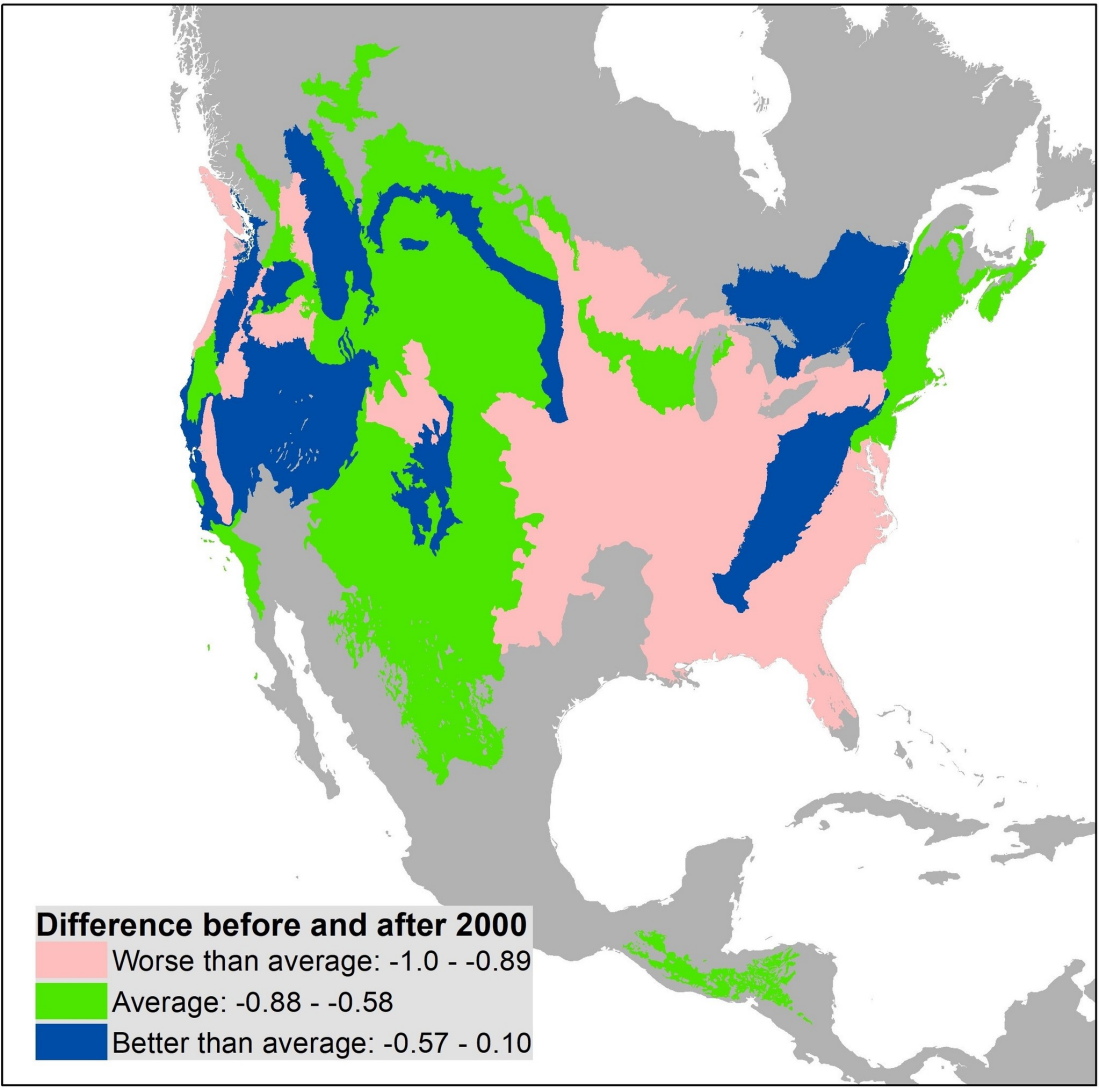
Change in the detection of *M*. *frenata* from museum and iNaturalist records between 1960–1999 (n = 736 records) and 2000–2020 (n = 843 records). For each era, detections are standardized as a proportion of all *M*. *frenata* records from a given ecoregion, with colors coded to highlight those doing worse than average (pink), within 15% points of average (green), and better than average (blue) as in Table 3.

Only regions with at least 10 records before 2000 are included. The average change for these 46 regions was -0.73. The table is sorted by proportional change and color coded to highlight those doing worse than average (pink), within 15% points of average (green), and better than average (blue) matching Fig 6.

### National trail camera survey

**Fig 7:**
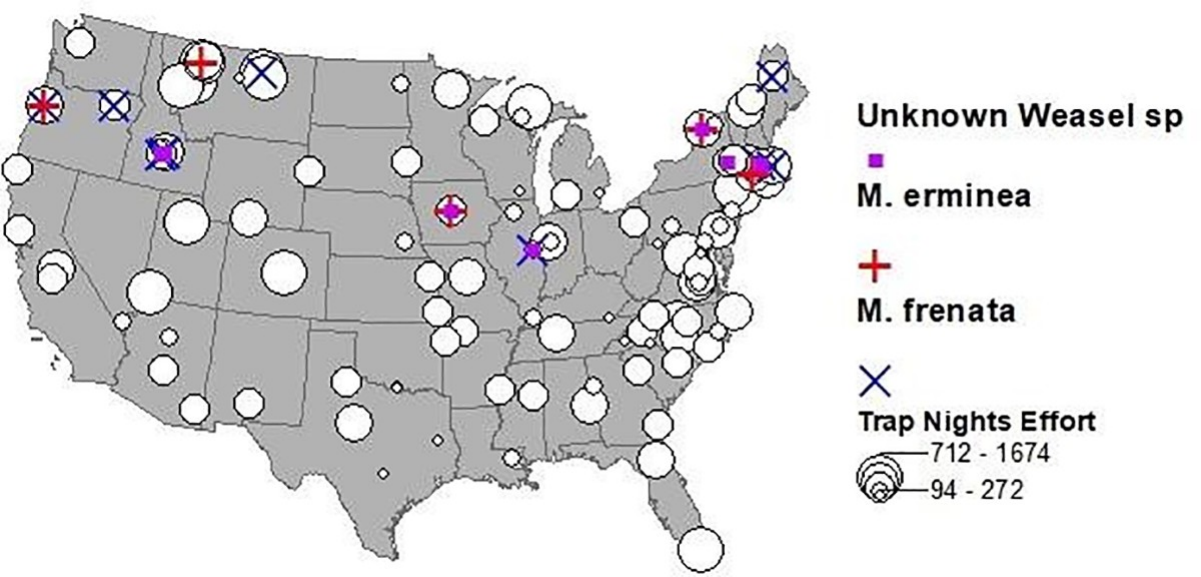
Results from the 2019 Snapshot USA mammal survey [21] which included 1509 trail cameras operated in 110 arrays in all 50 states (HI and AK not shown but did not detect weasels). Sample effort (trail camera nights) per array is shown by white dots while colored markers indicate which arrays detected at least one weasel.

In the 2019 Snapshot USA mammal survey, weasels were detected 51 times across 14 different camera arrays, including 7 detections of *M*. *erminea*, 17 of *M*. *frenata*, and 27 of weasels that could not be identified to species (Fig 7). All arrays that detected weasels were at or above 40^o^ latitude, with no detections at the 54 arrays farther south. At each array, weasels were only detected by one camera, although in 55% of these cases they were detected on multiple days by that camera.

## Discussion

Although weasels were historically harvested in large numbers, today they are infrequently harvested or detected in survey efforts across most of North America. Our analysis of long-term harvest data suggests weasel populations have declined precipitously in the last century. Weasel harvest has declined 2–6 orders of magnitude since the mid-1900s, and our analysis suggests this is not an artifact of declines in trapping effort alone. The most recent opportunistic observations by citizens (iNaturalist) allowed us to identify regions with many historical museum records but few or no such records in the last 20 years. This was especially the case for *M*. *frenata*. Finally, the recent systematic US-wide survey effort (Snapshot USA) confirmed weasels only in some northern sites.

Support for a negative effect of year on weasel harvest across many states and provinces suggests a decline in harvest over the past century that likely reflects an actual, rather than perceived, population decline across most of the range of each species. Reporting efforts varied over time among and within states and provinces, but there was a general decline in the number of fur trappers over time (S1 Fig) that somewhat paralleled a decline in weasel harvest. Nevertheless, in most states where we observed support for a positive effect of trapper number on weasel harvest, we also observed support for a negative effect of year. We did see exceptions to this trend of a negative effect of year (e.g., Connecticut, Newfoundland, Nova Scotia, Utah, Vermont) but data from those states/provinces were post-1980, which was after our identified widespread decline in the mid-1900s (Fig 1).

Pelt price is often used as a proxy for demand or trapper effort [33, 35, 36] and a decline in pelt price over time could have altered trapping practices such that trappers targeted other furbearer species. If so, most recent records might represent bycatch when targeting other species. Yet we did not observe consistent support for an effect of pelt price on annual total or per-trapper weasel harvest. This is somewhat expected given that weasels are consistently one of the lowest-valued pelts collected by trappers, and thus often captured as bycatch when targeting more high-value furbearers [15, 31]. It is also important to note that trapping practices have changed over the past century, particularly a decline in the use of leg-hold traps by trappers in favor of body-gripping traps that are less likely to capture weasels [31]. In a 2015 national survey of fur trappers in the US, weasels were among the least targeted furbearer species [41]. As such, a decline in harvest in the mid-20^th^ century might partially reflect a change in trapping practices. Nonetheless and overall, while harvest data are imperfect, our range-wide analysis supports previous assessments that harvest declines since the mid-20^th^ century represent a real decrease in weasel populations over time [18].

Unlike trapping data, museum and iNaturalist records were identified to species and georeferenced precisely, allowing us to consider distribution and abundance data at a finer scale. Because both collections are opportunistic, and because weasel records generally are sparse in museum collections, we were not able to document annual trends. Nevertheless, we were able to identify ecoregions where weasel populations were documented historically with some frequency (>10 museum specimens) but rarely or not documented in the last 20 years, despite substantial collection or observation of other small carnivores in those areas. While *M*. *erminea* and *M*. *nivalis* have fairly consistent records across most of their range, there were 21 ecoregions where records of *M*. *frenata* have declined precipitously, including a large swath of non-mountainous habitat in the central and southern portions of its US range, southern Great Lakes forests, and five non-contiguous western ecoregions. These contrast with northern and mountainous ecoregions where *M*. *frenata* is still consistently reported by museums and citizen scientists.

While it remains unclear what factors contributed to the wide-scale decline in weasel records over the past several decades, there are at least five potential hypotheses that deserve investigation. First, land-use change in the mid-20th century from smaller family farms to large-scale agriculture, and from native forest to intensive timber production, is a leading hypothesized driver of declines in small carnivores like eastern spotted skunks (*Spilogale putorius*) and weasels in North America [14, 32]. Historically, *M*. *frenata* was frequently reported as an agricultural pest to small farm owners by predating poultry, while also providing benefits by removing crop-consuming rodents [8]. A shift toward industrial row-crop agriculture and wide-scale use of rodenticides over the past century [42], along with associated declines in small mammal abundance and increasing habitat fragmentation, could thus have negatively impacted weasels across much of their historical range [15, 18, 31, 43]. Indeed in portions of Europe there is increasing concern of both the direct (on small mammal prey) and secondary effects of rodenticides in explaining declines of *Mustela* spp. [44, 45]. Second, predation by raptors and owls during winter [46–48] is widely viewed as the leading cause of mortality in North American weasel species. Thus, changes in forest management that facilitate owl predation (i.e., open understory) could negatively impact weasel populations similar to eastern spotted skunks [49]. Changes in mammalian predator guilds (e.g., expansion of red fox (*Vulpes vulpes*, [50]), as well as opossum (*Didelphis virginiana*, [51]) and raccoon [52]) could also be impacting weasels through inter-specific competition and predation [53]. Third, weasels are susceptible to multiple diseases (e.g., canine distemper, rabies, Aleutian disease, sylvatic plague) and disease has anecdotally been suggested as contributing to weasel decline [18, 31, 43]. In addition to the direct effects of disease on weasels, disease-related reductions in prey abundance could also influence weasel populations. For example, in a game reserve in Great Britain, a 10-fold reduction is stoat abundance was observed following an myxomatosis outbreak in rabbits [54]. Fourth, climate change has and is likely to continue to impact weasel populations in two primary ways. First, similar to the negative predicted impact of a warming climate and associated elevation shifts in forest communities on American marten (*Martes americana* [55, 56]), weasels could be negatively impacted by climate-induced shifts in habitat conditions and associated prey communities. Second, some weasel populations that turn white in the winter may be particularly vulnerable to climate change given the potential for coat color-habitat mismatch and associated elevated risk of predation [57, 58]. Finally, given the relatively high levels of historical harvest, historical overharvest cannot be ruled out as a cause of decline in portions of their range where harvest rates were particularly high.

While we used the best available data for our analysis, this exercise points out the need for better monitoring tools for weasel populations. Although fur harvest reports provided important historical context for our assessment, we think they are likely to be less useful moving forward given the relatively small number of weasels currently reported (particularly in more southern states), the shift in trapping techniques, and the overall decline in fur trapper numbers. In addition, weasel harvest typically is reported at the genus level, likely masking species-specific patterns. Observational citizen science data-reporting platforms like iNaturalist provide useful occurrence data and are growing rapidly, making them an important resource for conservation managers moving forward, although reporting is opportunistic in nature and thus has uneven spatial and temporal distribution. More systematic approaches like Snapshot USA illustrate the potential for trail cameras to be useful in gaining information on weasel distribution and habitat associations, although weasel detections were highly localized on these unbaited sites, with only one camera in an array detecting weasels. The placement, orientation and model of camera influence the detection probability of small carnivores [59, 60] and direct evaluation of each of these factors (in addition to use of baits) on weasel detection probability needs to be investigated. Recently in Europe, baited trail cameras within enclosed boxes specifically designed for surveying weasels showed increased detection probability and limited non-target animal photographs [13, 61]. In New Zealand, where extensive research has taken place on invasive *M*. *erminea*, recent comparative investigations have found that use of artificial nests and baited trail cameras improve stoat detection compared to traditional footprint-tracking tunnels [62]. The success of these new techniques suggests opportunities exist to develope weasel-specific, baited monitoring approaches in North America to enable more nuanced understanding of the broad patterns reported here.

Given that weasel species are ranked as taxa of low conservation concern across two thirds of range states and provinces (Table 1), our data suggest the need to revisit the conservation status of weasel species across North America. Our findings highlight the need for development of a long-term, weasel-specific monitoring program that is replicated both spatially and temporally, such that it can provide information on distributions and trends both locally and across the range of each species. While the wide distribution, cryptic nature, and scale of possible declines make such a task daunting, advancements in monitoring techniques and collaboration could make it possible. A potential model is the Eastern Spotted Skunk Cooperative Study Group, which was formed in 2015 to enhance communication, identify management priorities and develop collaborative monitoring initiatives and research on the species [63]. We believe that taking steps now could pay dividends by avoiding future costly recovery actions should these observed declines be further substantiated.

By identifying a decline in a formerly abundant genus of small carnivores across North America, our findings echo recent calls to expand investigations into the conservation need of small carnivores [4]. In particular, there should be serious concern for small carnivores in more species-rich and data deficient portion of globe such as Southeast Asia, sub-Saharan Africa, and Madagascar where small carnivores are at greatest risk of extinction [4]. Further, given such underappreciated declines can take place on a continent with relatively long-term monitoring data for carnivores, we encourage similar reviews of small carnivores globally.

## Supporting information

S1 Fig Weasel pelt price over time in North America from states and provinces included in harvest analyses. All values reported in US dollars and adjusted for consumer price index to 2017 values. See Table 1 for state/province abbreviations.
(DOCX)

S2 Fig Number of trappers reported for North American states and provinces used in harvest analyses. See Table 1 for state/province abbreviations.
(DOCX)

S1 Table State- and province-specific analysis of annual trend in weasel harvest from 1920–2017. Assessing the effects of previous year pelt price and number of trappers on annual trends in harvest and per capita harvest (Harvest ~ Year + lagPelt, Harvest ~ Year + lagPelt + Trappers, adjHarvest = Year + Pelt). Beta estimates and 95% confidence intervals (CI) of year, previous year pelt price, and number of trappers from linear regression models. See Table 1 for state/province abbreviations.
(DOCX)

S1 File (XLSX)
