# Supplementary material for: Tracking the decline of weasels in North America
Source: PLoS One. 2021 Jul 21;16(7):e0254387. doi: 10.1371/journal.pone.0254387 (PMC8294569; doi:10.1371/journal.pone.0254387)

S1 Fig. Weasel pelt price over time in North America from states and provinces included in harvest analyses. All values reported in US dollars and adjusted for consumer price index to 2017 values. See Table 1 for state/province abbreviations.


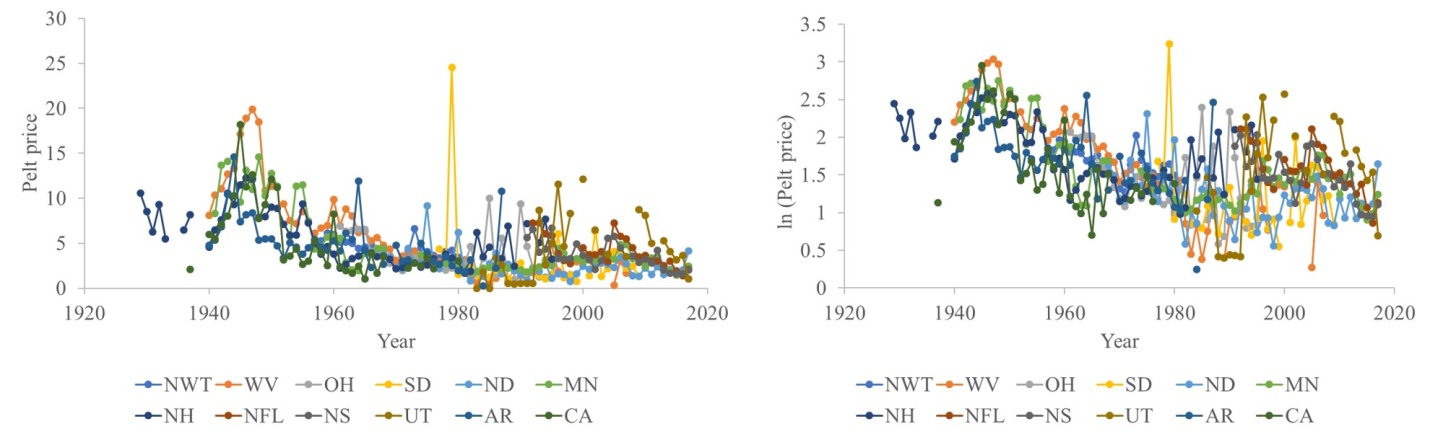

Supplement: S1 Fig — All values reported in US dollars and adjusted for consumer price index to 2017 values. See Table 1 for state/province abbreviations. (DOCX) [file pone.0254387.s001.docx]
