# Supplementary material for: Tracking the decline of weasels in North America
Source: PLoS One. 2021 Jul 21;16(7):e0254387. doi: 10.1371/journal.pone.0254387 (PMC8294569; doi:10.1371/journal.pone.0254387)

S2 Fig. Number of trappers reported for North American states and provinces used in harvest analyses. See Table 1 for state/province abbreviations.


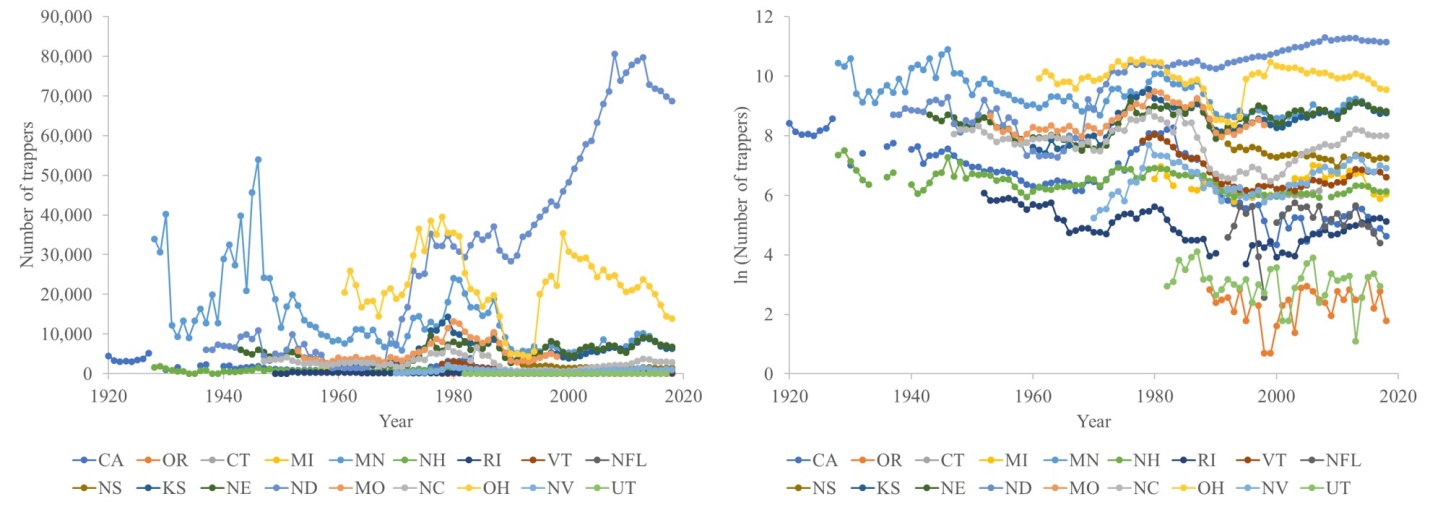

Supplement: S2 Fig — See Table 1 for state/province abbreviations. (DOCX) [file pone.0254387.s002.docx]
