# Supplementary material for: Tracking the decline of weasels in North America
Source: PLoS One. 2021 Jul 21;16(7):e0254387. doi: 10.1371/journal.pone.0254387 (PMC8294569; doi:10.1371/journal.pone.0254387)
Supplement: S1 Table — Assessing the effects of previous year pelt price and number of trappers on annual trends in harvest and per capita harvest (Harvest ~ Year + lagPelt, Harvest ~ Year + lagPelt + Trappers, adjHarvest = Year + Pelt). Beta estimates and 95% confidence intervals (CI) of year, previous year pelt price, and number of trappers from linear regression models. See Table 1 for state/province abbreviations. (DOCX) [file pone.0254387.s003.docx]

S1 Table. State- and province-specific analysis of annual trend in weasel harvest from 1920-2017. Assessing the effects of previous year pelt price and number of trappers on annual trends in harvest and per capita harvest (Harvest ~ Year + lagPelt, Harvest ~ Year + lagPelt + Trappers, adjHarvest = Year + Pelt). Beta estimates and 95% confidence intervals (CI) of year, previous year pelt price, and number of trappers from linear regression models. See Table 1 for state/province abbreviations.

| Model | State | Year | 95% CI | *p* | lagPelt | 95% CI | *p* | Trappers | 95% CI | *p* |
| --- | --- | --- | --- | --- | --- | --- | --- | --- | --- | --- |
| Harvest ~ Year + lagPelt | AR | -2.08 | -2.64, -1.52 | <0.001 | 0.15 | -0.26, 0.56 | 0.477 |  |  |  |
|  | NWT | -1.35 | -1.98, -0.72 | <0.001 | 0.33 | -0.23, 0.88 | 0.263 |  |  |  |
|  | SD | -1.49 | -2.55, -0.42 | 0.011 | 0.69 | 0.20, 1.19 | 0.011 |  |  |  |
|  | WV | -1.91 | -2.48, -1.35 | <0.001 | 0.07 | -0.30, 0.43 | 0.726 |  |  |  |
| Harvest ~ Year + lagPelt + Trappers | CA | -1.34 | -1.84, -0.84 | <0.001 | 0.21 | -0.04, 0.45 | 0.105 | 2.62 | 1.63, 3.61 | <0.001 |
|  | MN | -0.09 | -0.30, 0.12 | 0.393 | 0.76 | 0.57, 0.94 | <0.001 | 1.43 | 0.73, 2.12 | <0.001 |
|  | NH | -0.83 | -1.08, -0.57 | <0.001 | -0.08 | -0.33, 0.17 | 0.527 | 0.93 | -0.24, 2.11 | 0.125 |
|  | NL | -0.15 | -0.63, 0.34 | 0.558 | 0.33 | 0.09, 1.36 | 0.014 | 0.58 | 0.03, 1.14 | 0.052 |
|  | ND | -2.30 | -3.41, -1.18 | <0.001 | 0.76 | 0.11, 1.40 | 0.027 | 0.48 | -1.96, 2.92 | 0.704 |
|  | NS | -0.01 | -1.41, 1.38 | 0.986 | 0.73 | 0.09, 1.36 | 0.035 | -2.18 | -7.89, 3.35 | 0.461 |
|  | OH | -2.59 | -3.18, -2.00 | <0.001 | 0.16 | -0.11, 0.42 | 0.265 | 2.51 | 1.62, 3.41 | <0.001 |
|  | UT | 0.38 | -0.34, 1.09 | 0.311 | -0.06 | -0.30, 0.18 | 0.604 | 2.89 | 1.74, 4.03 | <0.001 |
| adjHarvest ~ Year + lagPelt | CA | -0.54 | -0.75, -0.32 | <0.001 | 0.12 | 0.01, 0.22 | 0.031 |  |  |  |
|  | MN | 0.01 | -0.04, 0.06 | 0.789 | 0.02 | 0.15, 0.25 | <0.001 |  |  |  |
|  | NH | -0.32 | -0.42, -0.22 | <0.001 | 0.05 | -0.14, 0.05 | 0.338 |  |  |  |
|  | NL | -0.24 | -0.62, 0.13 | 0.220 | -0.06 | -0.23, 0.12 | 0.527 |  |  |  |
|  | ND | -0.62 | -0.78, -0.46 | <0.001 | 0.21 | 0.04, 0.37 | 0.019 |  |  |  |
|  | NS | 0.09 | -0.06, 0.24 | 0.259 | 0.11 | 0.02, 0.19 | 0.028 |  |  |  |
|  | OH | -0.84 | -1.00, -0.69 | <0.001 | 0.01 | -0.07, 0.09 | 0.855 |  |  |  |
|  | UT | 0.58 | -0.19, 1.36 | 0.152 | -0.124 | -0.38, 0.15 | 0.390 |  |  |  |
